# A Case of Umbilical Hernia

**Published:** 1852-02

**Authors:** M. Shepherd

**Affiliations:** Payson, Ills.


### WESTERN JOURNAL

OF

### MEDICINE AND SURGERY.

FEBRUARY, 1852.

---

ART. I.—*A Case of Umbilical Hernia.* By M. SHEPHERD, M. D., of Payson, Ills.

It has been remarked "that he who wishes to make himself well acquainted with the pathology of hernia, and with all the circumstances which are constantly interfering to modify its treatment, should not miss a single opportunity which may present itself of observing every case which may fall in his way. It may be stated as a great probability that, with every fresh case, something new will be learned. Hence we consider a faithful and accurate report of all individual cases of hernia, to be most especially useful."

This is our apology for offering the following case to the readers of the Journal, which we hope may not prove uninteresting to the young practitioner.

On the 27th of August, 1851, I was requested to visit

Mrs. S——, a corpulent lady of sixty years, the mother of ten or twelve children. I found her suffering from umbilical hernia, with a tumor nearly as large as a child's head. I was informed that the tumor had existed for twenty-five years, or more, though not so large all that time. A portion of the bowel, it seemed, from the history she gave, had passed through the ring a number of times since the tumor was first formed, always attended with severe pain for from six to twelve hours. She had been in the habit, on such attacks, of taking a dose of castor oil, and assuming a recumbent position, which was maintained until she obtained relief—never having called a physician for this disease before. On this occasion, however, she had not been so fortunate, having suffered four days before I was called. I should have remarked that, the old lady's general health had been such as to confine her to her bed a greater part of the time, for three weeks previous to this attack. I resorted to all the common means for reducing hernia without avail.

As there was no evidence of strangulation, I ordered a dose of castor oil, to be assisted at the end of three hours by an enema, which was to be repeated *pro re nata* until her bowels should move—and left her for the night.—Early the next morning I was summoned in haste to see her again. The means resorted to during the night had failed to evacuate the bowels, and about day-light she commenced vomiting bilious and stercoraceous matter.—Her pulse was small and thread-like, she had hiccups and a cold, clammy sweat. I proposed an operation immediately as the only means of affording relief. To this, the old lady and a portion of her friends objected; as she was so old it was thought she might as well die with the disease as to be subjected to the knife. I did not insist upon operating, believing it best to allow them to decide upon that matter themselves. The decision was that all of her children should be called in, first, and an expression obtained from them, which would require till 2 or 3 o'clock, P. M., at which time, if they agreed that she should undergo an operation, I should be called again. As I was professionally detained in another part of the county, I did not receive intelligence from her until late in the afternoon, and the operation was not commenced until candle-lighting. An incision was commenced in the mesial line about midway of the tumor and extended toward its lower aspect, another, commencing at the same point was extended laterally making but one flap. I was careful in dissecting through the adipose tissue, and at first was somewhat surprised that I discovered no hernial sac. After a moment's reflection, however, in regard to the length of time that the tumor had existed, I was satisfied that the sac had been absorbed. The portion of the bowel that was strangulated was not large, but presented a very dark reddish appearance. Finding it impossible to reduce that without cutting the stricture, I introduced a probe-pointed bistoury, guided by the fore-finger of the left hand, at the lower edge of the ring, and cut a very little downwards, when the bowel passed in, readily. In attempting to return the omentum, I found that the entire base of the tumor was firmly attached to the outer wall of the abdomen. From the length of time that the attachment must have existed, it was not deemed advisable to attempt to break it up, and it being very vascular, I could not, as has been suggested by some authors, remove it by the knife. As the strangulation was relieved, I at once concluded to close the integuments over the tumor as it then existed and await the result. This was done, a compress and a bandage applied, and to my entire satisfaction a greater part of the wound united by the first intention. Notwithstanding the oppressively hot weather during the month of September, the old lady, under the use of wine and a generous diet, improved very much. But before she had entirely recovered, during the month of October, she had an attack of dysentery, which prevailed epidemically at that time, and was carried off in a few days.

Was the course pursued the best under the existing circumstances? or should the attachment have been broken up? or the omentum removed by the knife?

Payson, Ills., January, 1852.

